# An Integrated Analysis of miRNA and mRNA Expressions in Non-Small Cell Lung Cancers

**DOI:** 10.1371/journal.pone.0026502

**Published:** 2011-10-27

**Authors:** Lina Ma, Yanyan Huang, Wangyu Zhu, Shiquan Zhou, Jihang Zhou, Fang Zeng, Xiaoguang Liu, Yongkui Zhang, Jun Yu

**Affiliations:** 1 Joint Laboratory of Immunogenomics, Zhoushan Hospital-BIGCAS, Zhejiang, People's Republic of China; 2 CAS Key Laboratory of Genome Sciences and Information, Beijing Institute of Genomics, Chinese Academy of Sciences, Beijing, People's Republic of China; Karolinska Institutet, Sweden

## Abstract

Using DNA microarrays, we generated both mRNA and miRNA expression data from 6 non-small cell lung cancer (NSCLC) tissues and their matching normal control from adjacent tissues to identify potential miRNA markers for diagnostics. We demonstrated that hsa-miR-96 is significantly and consistently up-regulated in all 6 NSCLCs. We validated this result in an independent set of 35 paired tumors and their adjacent normal tissues, as well as their sera that are collected before surgical resection or chemotherapy, and the results suggested that hsa-miR-96 may play an important role in NSCLC development and has great potential to be used as a noninvasive marker for diagnosing NSCLC. We predicted potential miRNA target mRNAs based on different methods (TargetScan and miRanda). Further classification of miRNA regulated genes based on their relationship with miRNAs revealed that hsa-miR-96 and certain other miRNAs tend to down-regulate their target mRNAs in NSCLC development, which have expression levels permissive to direct interaction between miRNAs and their target mRNAs. In addition, we identified a significant correlation of miRNA regulation with genes coincide with high density of CpG islands, which suggests that miRNA may represent a primary regulatory mechanism governing basic cellular functions and cell differentiations, and such mechanism may be complementary to DNA methylation in repressing or activating gene expression.

## Introduction

Currently, non-small cell lung cancer (NSCLC) is the leading cause of cancer death in the world[1]. Surgical resection is mainly performed in early-stage cases and is also effective, but its effect is limited for patients with locally advanced cancers because of the high rate of distant metastasis and recurrence. Thus, chemotherapy and radiation therapy, as well as a combination of both, are always performed in the treatment of patients with locally advanced cancers. However, the 5-year survival rate of lung cancer patients decreases dramatically with clinical stages of the disease, for instance, from an estimated 61% for patients with the stage IA disease to 38% for patients with the stage IB disease. Other stage-related 5-year survival rate of the disease are 34% for patients with IIA disease, 13% for patients with stage IIIA disease, 5% for patients with stage IIIB disease, and 1% for patients with stage IV [2], [3], [4], [5]. Most lung cancer patients are only diagnosed after tumors have spread beyond their primary sites, and this is an important reason for the poor outcome of lung cancer treatments. In addition, variation analysis among a variety of cancers suggested that lung cancer has larger number of mutated genes than other cancers [6]. Therefore, the discovery of effective diagnostic markers and interrogating mechanisms of lung cancer development, are both necessary for improving the survival rate of lung cancer patients.

miRNAs are short non-coding RNAs (∼22 nt long) that bind complementary sequences in target mRNAs, resulting in their selective degradation or selective inhibition of translation. Therefore, through regulating their target genes, miRNAs are known to be involved in a wide range of biological functions, such as cellular proliferation, differentiation, and apoptosis [7], [8]. Aberrant miRNAs expression has been reported to cause diseases, such as cancers [9], Alzheimer's disease [10], heart diseases [11], spinal motor neuron anomalies [12], and etc.. In NSCLC, it has been suggested that miR-31 may act as an oncogenic miRNA by repressing tumor suppressors: LATS2 and PPP2R2A [13], and expression of hsa-miR-205 has been suggested to be able to distinguish squamous from nonsquamous non-small-cell lung carcinoma [14]. In addition, evidences show that high hsa-mir-155 and low hsa-let-7a-2 expression correlated with poor survival of lung cancer patients [15], and has-miRNA-126 may promote NSCLC cells apoptosis induced by irradiation through the PI3K-Akt pathway [16]. miRNAs are now emerging as highly tissue-specific biomarkers for discriminate cancers from noncancerous tissue, and different cancer types, as well as different prognostic results [15], [17].

mRNA profiling have revealed that transcriptional abnormality of many genes are responsible for the development of NSCLC [18], [19], and differential expression of miRNAs also have been detected between lung cancer tissues and the adjacent normal tissues [15], [20]. In the present study, we used microarrays to measure the expression levels of miRNAs and mRNAs simultaneously in paired NSCLC and their adjacent normal tissues to investigate possible roles of miRNAs. Our analyses are both integrated and rather deep in search for possible candidate miRNAs and their mRNA targets for further investigations and clinical applications.

## Results

### Differentially expressed mRNAs in NSCLC tissues compared with their paired normal tissues

We performed gene expression profiling for paired tumor-normal tissue samples from 6 NSCLC patients and investigated 34,694 genes/transcripts. We defined 581 up-regulated genes and 1, 297 down-regulated genes which were consistently up- or down-regulated in all 6 tumor tissues compared with the adjacent normal tissues (Table S2 and Table S3). Among differentially expressed genes, we identified 2 significantly up-regulated and 22 significantly down-regulated genes at a False Discovery Rate (FDR) of 0.1 (Table S2 and Table S3). We subsequently performed GO enrichment analysis using hypergeometric test and multiple test adjustment method BH through WebGestalt2 [21] and defined the enriched pathways at a *p* value of <0.001. The hypergeometric distribution is a discrete probability distribution that describes the number of successes in a sequence of *n* draws from a finite population without replacement, just as the binomial distribution describes the number of successes for draws with replacement. Hypergeometric test helps to find which GO terms are overrepresented in a large gene list. According to hypergeometric test results, the cell cycle M-phase and DNA metabolic process were predominantly activated, whereas pathways involved in immune functions were significantly repressed in NSCLC (Table 1). In addition, up-regulated genes were predominantly enriched in “chromosome” as opposed to down-regulated genes that were predominantly enriched in “cell membrane” (Table 2).

**Table 1:** Enriched GO terms for biological process based on variable genes in lung cancer tissues when compared with their adjacent normal tissues.

Class	GO terms	GO number	Statistic*
**Up-regulated**	DNA metabolic process	GO:0006259	C = 552;O = 27;E = 9.68;R = 2.79;rawP = 1.48e-06;adjP = 0.0008
**Up-regulated**	M phase	GO:0000279	C = 370;O = 21;E = 6.49;R = 3.24;rawP = 2.35e-06;adjP = 0.0008
**down-regulated**	immune response	GO:0006955	C = 750;O = 101;E = 42.54;R = 2.37;rawP = 1.51e-16;adjP = 2.91e-13
**down-regulated**	immune system process	GO:0002376	C = 1066;O = 124;E = 60.46;R = 2.05;rawP = 3.98e-15;adjP = 3.84e-12
**down-regulated**	defense response	GO:0006952	C = 657;O = 89;E = 37.26;R = 2.39;rawP = 8.14e-15;adjP = 5.23e-12
**down-regulated**	inflammatory response	GO:0006954	C = 359;O = 60;E = 20.36;R = 2.95;rawP = 2.82e-14;adjP = 1.36e-11
**down-regulated**	response to wounding	GO:0009611	C = 560;O = 75;E = 31.76;R = 2.36;rawP = 2.23e-12;adjP = 8.60e-10
**down-regulated**	response to stress	GO:0006950	C = 1696;O = 147;E = 96.19;R = 1.53;rawP = 5.52e-08;adjP = 1.33e-05
**down-regulated**	innate immune response	GO:0045087	C = 176;O = 30;E = 9.98;R = 3.01;rawP = 5.52e-08;adjP = 1.33e-05
**down-regulated**	response to external stimulus	GO:0009605	C = 904;O = 91;E = 51.27;R = 1.77;rawP = 4.51e-08;adjP = 1.33e-05
**down-regulated**	immune effector process	GO:0002252	C = 200;O = 31;E = 11.34;R = 2.73;rawP = 3.06e-07;adjP = 6.56e-05
**down-regulated**	regulation of immune system process	GO:0002682	C = 362;O = 45;E = 20.53;R = 2.19;rawP = 5.72e-07;adjP = 0.0001
**down-regulated**	leukocyte mediated immunity	GO:0002443	C = 126;O = 22;E = 7.15;R = 3.08;rawP = 2.14e-06;adjP = 0.0002
**down-regulated**	actin cytoskeleton organization	GO:0030036	C = 257;O = 35;E = 14.58;R = 2.40;rawP = 1.28e-06;adjP = 0.0002
**down-regulated**	positive regulation of immune system process	GO:0002684	C = 229;O = 32;E = 12.99;R = 2.46;rawP = 2.08e-06;adjP = 0.0002
**down-regulated**	lymphocyte activation	GO:0046649	C = 272;O = 36;E = 15.43;R = 2.33;rawP = 1.80e-06;adjP = 0.0002
**down-regulated**	T cell activation	GO:0042110	C = 194;O = 29;E = 11.00;R = 2.64;rawP = 1.57e-06;adjP = 0.0002
**down-regulated**	leukocyte activation	GO:0045321	C = 324;O = 41;E = 18.38;R = 2.23;rawP = 1.15e-06;adjP = 0.0002
**down-regulated**	actin filament-based process	GO:0030029	C = 274;O = 36;E = 15.54;R = 2.32;rawP = 2.14e-06;adjP = 0.0002
**down-regulated**	adaptive immune response	GO:0002250	C = 113;O = 20;E = 6.41;R = 3.12;rawP = 4.97e-06;adjP = 0.0005
**down-regulated**	adaptive immune response based on somatic recombination of immune receptors built from immunoglobulin superfamily domains	GO:0002460	C = 112;O = 20;E = 6.35;R = 3.15;rawP = 4.31e-06;adjP = 0.0005
**down-regulated**	lymphocyte mediated immunity	GO:0002449	C = 106;O = 19;E = 6.01;R = 3.16;rawP = 7.04e-06;adjP = 0.0007
**down-regulated**	positive regulation of biological process	GO:0048518	C = 1865;O = 148;E = 105.77;R = 1.40;rawP = 9.03e-06;adjP = 0.0008
**down-regulated**	cell activation	GO:0001775	C = 366;O = 42;E = 20.76;R = 2.02;rawP = 1.05e-05;adjP = 0.0009

*The alst column lists the number of reference genes in the category (C), number of genes in the gene set and also in the category (O), expected number in the category (E), Ratio of enrichment (R), *p* value from hypergeometric test (rawP), and *p* value adjusted by the multiple test adjustment (adjP).

**Table 2:** Enriched GO terms for cellular components based on variable genes in lung cancer tissues when compared with their adjacent normal tissues.

Class	GO terms	GO number	Statistic*
**Up-regulated**	chromosome	GO:0005694	C = 454;O = 26;E = 8.45;R = 3.08;rawP = 3.99e-07;adjP = 5.35e-05
**Up-regulated**	chromosomal part	GO:0044427	C = 378;O = 23;E = 7.03;R = 3.27;rawP = 6.77e-07;adjP = 5.35e-05
**Up-regulated**	chromosome, centromeric region	GO:0000775	C = 120;O = 11;E = 2.23;R = 4.93;rawP = 1.48e-05;adjP = 0.0008
**Down-regulated**	plasma membrane part	GO:0044459	C = 1918;O = 169;E = 104.66;R = 1.61;rawP = 7.90e-11;adjP = 2.21e-08
**Down-regulated**	integral to plasma membrane	GO:0005887	C = 1183;O = 112;E = 64.55;R = 1.74;rawP = 4.46e-09;adjP = 6.24e-07
**Down-regulated**	plasma membrane	GO:0005886	C = 3650;O = 269;E = 199.17;R = 1.35;rawP = 1.34e-08;adjP = 9.38e-07
**Down-regulated**	intrinsic to plasma membrane	GO:0031226	C = 1206;O = 112;E = 65.81;R = 1.70;rawP = 1.30e-08;adjP = 9.38e-07
**Down-regulated**	membrane part	GO:0044425	C = 6381;O = 417;E = 348.19;R = 1.20;rawP = 7.79e-07;adjP = 3.64e-05
**Down-regulated**	membrane	GO:0016020	C = 7186;O = 462;E = 392.12;R = 1.18;rawP = 7.05e-07;adjP = 3.64e-05
**Down-regulated**	I-kappaB/NF-kappaB complex	GO:0033256	C = 4;O = 4;E = 0.22;R = 18.33;rawP = 8.81e-06;adjP = 0.0004
**Down-regulated**	lytic vacuole	GO:0000323	C = 206;O = 27;E = 11.24;R = 2.40;rawP = 2.13e-05;adjP = 0.0006
**Down-regulated**	intrinsic to membrane	GO:0031224	C = 5451;O = 355;E = 297.44;R = 1.19;rawP = 1.80e-05;adjP = 0.0006
**Down-regulated**	lysosome	GO:0005764	C = 206;O = 27;E = 11.24;R = 2.40;rawP = 2.13e-05;adjP = 0.0006
**Down-regulated**	cell-substrate adherens junction	GO:0005924	C = 100;O = 17;E = 5.46;R = 3.12;rawP = 2.67e-05;adjP = 0.0007

*The last column lists the number of reference genes in the category (C), number of genes in the gene set and also in the category (O), expected number in the category (E), Ratio of enrichment (R), *p* value from hypergeometric test (rawP), and *p* value adjusted based on the multiple test adjustment (adjP).

### Differentially expressed miRNAs in NSCLC tissues compared with their paired normal tissues

**Figure 1:**
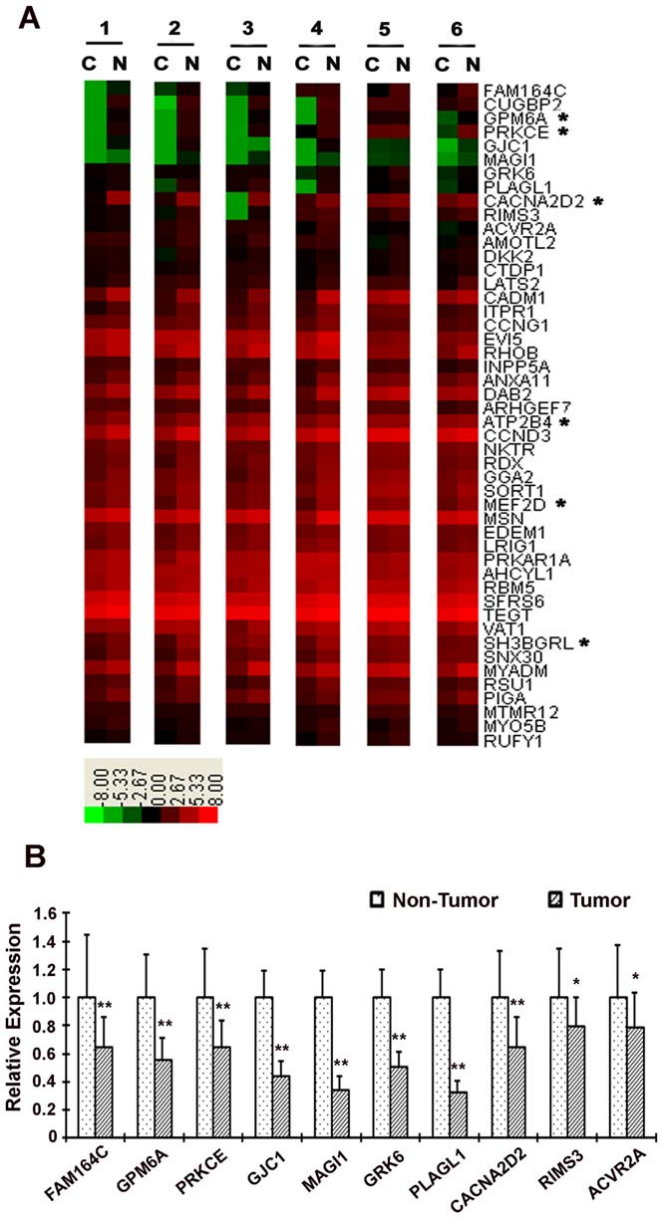
Down-regulated target candidates of hsa-miR-96 in NSCLC. (A) Microarray results of the Down-regulated target candidates of hsa-miR-96 in NSCLC. We assayed 6 paired NSCLC vs. normal tissue. C and N stand for cancer and adjacent normal tissue, respectively. The asterisk marks genes belong to the correlated group and the remaining genes are grouped into the anti-correlated group according to the expression relationship of these genes with their regulatory miRNAs. (B) Validation of microarray results by qRT-PCR. We selected 10 down-regulated target candidates of hsa-miR-96 and performed qRT-PCR experiments for the validation of relative mRNA expression in reference to glyceraldehyde-3-phosphate dehydrogenase (GAPDH). The relative expression values are the means ± SE. *, *P*<0.05 by *t* test; **, *P*<0.001 by *t* test.

**Figure 2:**
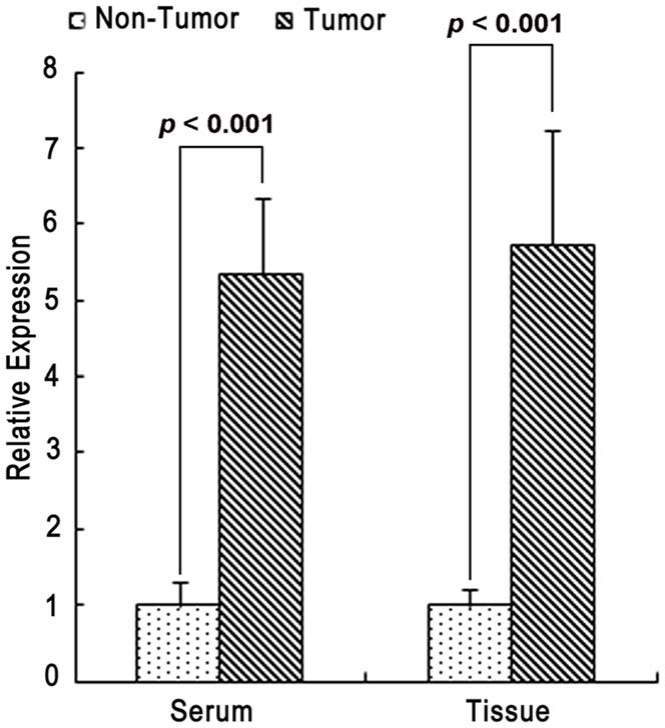
Quantitative RT-PCR analysis of hsa-miR-96 expression. Two groups of comparisons were performed: (1) tumor vs. adjacent normal lung tissues, and (2) cancer serum vs. non-cancer serum. Relative hsa-miR-96 expression was determined in reference to an internal U6 snRNA control. Relative expression values are the normalized mean ± SE.

We performed miRNA expression profiling using the same 6 paired samples of the primary tumor and its adjacent normal tissue. We obtained 25 up-regulated and 24 down-regulated miRNAs which were consistently up- or down-regulated in all 6 tumor tissues compared with the adjacent normal tissues before significance test (Table S4 and Table S5). Based on a more stringent analysis, we obtained a single significantly up-regulated miRNA, hsa-miR-96, at a False Discovery Rate (FDR) of 0.1. We further examined the expression level of its potential mRNA targets (These targets have conserved miRNA binding sites among vertebrates or mammals and were predicted using TargetScan): 13 out of 728 total candidate target genes (Table S2 and Table S6) (account for 2.24% of the 581 up-regulated genes) were up-regulated and 48 out of the total (Table S3 and Table S6) (account for 3.70% of the 1, 297 down-regulated genes) were down-regulated (Figure 1A). We then validated hsa-miR-96 expression based on quantitative RT-PCR in an independent set of 35 NSCLC and their serum samples (for the sera, expression values were normalized to normal people without cancer history or other illness at that time), and found hsa-miR-96 was significantly up-regulated in both tissue and serum samples from NSCLC patients (Figure 2). Therefore, hsa-miR-96 may be an important factor to contribute to NSCLC development and may have great potential to be used for diagnosing.

As miRNAs always repress the expression of the target genes, the 48 down-regulated targets of hsa-miR-96 may be most likely to contribute to NSCLC development through hsa-miR-96 regulation. We selected 10 down-regulated targets of hsa-miR-96 to validate their mRNA expression levels using qRT-PCR in 20 NSCLC and found that all of them were significantly down-regulated in tumor tissues when compared with the adjacent normal tissues (Figure 1B). To investigate how these 48 down-regulated potential mRNA targets may affect the development of NSCLC, we classified them based on GO terms, and found that these genes were involved in a variety of basic biological process, including metabolism, biological regulation, cell communication, developmental process, immune system, and etc. (Figure S2).

### Classification of mRNAs based on their relationship with miRNA regulation

To characterize how miRNAs may regulate their target mRNAs, we performed an extensive analysis on the correlated expression of miRNAs and their target mRNAs. miRNAs are expressed as long precursor RNAs that are processed by a cellular nuclease, Drosha, before being transported by an Exportin-5-dependent mechanism into the cytoplasm [22], [23]. Once in the cytoplasm, miRNAs are cleaved further by the enzyme DICER [24], [25], and this results in 17–24 nt miRNAs that are associated with a cellular complex that is similar to the RNA-induced silencing complex that participates in RNA interference. However, miRNAs mainly regulate mRNA translation, whereas siRNAs direct RNA destruction via the RNA interference (RNAi) pathway [8], [26]. Therefore, the abundance of miRNAs may mainly depend on its original expression and not imported exogenously. In addition, several lines of evidence suggest that elevation of miRNA expression lead to down-regulation of the target genes, and reduction of miRNA expression lead to up-regulation of the target genes [13], [27]. We therefore hypothesize that the effect of miRNAs on their target mRNAs is expression level-dependent. We used a novel measure to evaluate miRNA regulation, termed *regulation value*. We first assume that a regulation value of a miRNA is positively correlated with its expression level. Since a miRNA always has more than one target genes [28], we then assume that a regulation value of a given miRNA is negatively correlated with the number of its targets. Therefore, we define a regulation value for miRNA as the deviation in expression level for a given miRNA divided by the number of expressed mRNA targets.

During cancer development, the regulation may vary from case to control for a given miRNA and, therefore, regulation value of a given miRNA equals to the variation of miRNA expression divided by the number of expressed targets. When the expression of a miRNA is down-regulated in cancers, its regulation value becomes negative if the number of its expressed targets is not changed significantly. For a target mRNA, its regulation value is the sum of all regulation values from its regulatory miRNA. Based on these definitions, we classified all differentially expressed genes in cancers into different groups based on their correlations—correlated or anti-correlated with miRNA regulation. Before doing this, we predicted the potential target genes of these consistently up- or down-regulated miRNAs.

We used three methods to predict the potential targets of miRNAs. The software TargetScan focuses more on miRNA seed (2-8nt in the 5′ region of mature miRNA), as evidence suggests the importance of this region for miRNA target recognition [29], [30], [31], [32]. In addition, the sequence conservation of target sites has been considered as an important feature to reduce false-positive rate [30]. We therefore predicted potential miRNA targets using TargetScan and conserved target sites among vertebrates or mammals using *P*_CT_ method of TargetScan [33]. However, seed sites do not always confer repression, and the software miRanda also takes into consideration of other region of miRNA in addition to seed sites [34]. We then used miRanda as another alternative method to predict miRNA targets. The two software were frequently used to predict miRNA targets, and in addition, both of them can be used to predict targets of novel miRNAs as the illuminia miRNA microarray "humanMI_V2" contains many novel miRNAs that are generated from next-generation sequencing efforts worldwide. We obtained 16,160 conserved miRNA-target pairs among vertebrates or mammals using *P*_CT_ method of TargetScan (Table S6), 70,320 miRNA-target pairs by using TargetScan without considering the conservation condition (Table S8), and 77,988 miRNA-target pairs by using miRanda (Table S10). We observed that 48,841 miRNA-target pairs were present in both TargetScan and miRanda results (69.46% of TargetScan results, 62.63% of miRanda results), and 12,403 miRNA-target pairs of conserved TargetScan results were common with miRanda results (76.75% of conserved TargetScan results). Therefore, there were about 70% prediction results that were common in the two different methods, and conserved prediction results of TargetScan exhibite a larger proportion of shared genes.

**Figure 3:**
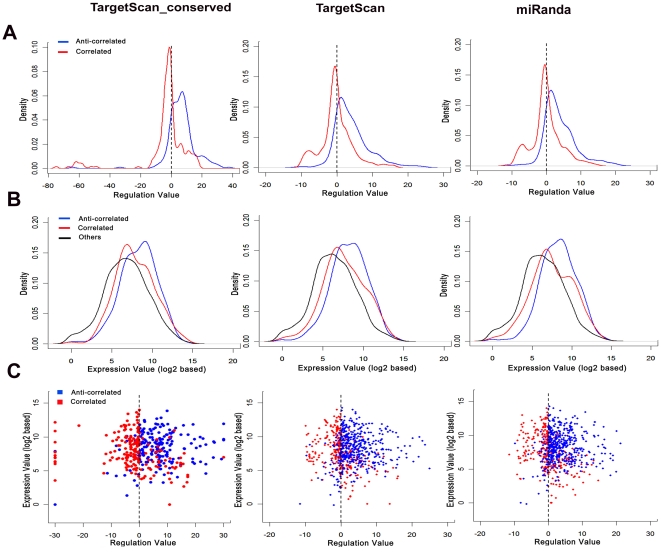
miRNA regulation analysis. We classified all expression-variable mRNAs during lung cancer development based on their relationship with miRNAs. The results are classified into correlated, anti-correlated, and others (no correlation). The correlated mRNAs show correlated expressions to their regulatory miRNA expressions and the anti-correlated mRNAs are not. Three different methods were used to predict potential miRNA targets: “Conserved” are those genes that have conserved miRNA binding sites among vertebrates or mammals, and these genes were predicted by using the *P*_CT_ method of TargetScan; “TargetScan” are those genes that are predicted by using a perl script of TargetScan without considering conservation. “miRanda” are those genes that are predicted potential targets based on miRanda v3.3a on Linux platform. (A) Gene distribution of the correlated and anti-correlated mRNAs was plotted based on their miRNA regulation values. (B) Gene distribution of the three groups of mRNAs was plotted based on their expression values. The expression value was defined by referencing that of the adjacent normal tissue (log2). (C) The relationship between gene expression and miRNA regulation.

We firstly used the conserved prediction results to classify mRNAs based on their relationship with miRNA regulation. In particular, we defined 197 and 190 genes as anti-correlated and correlated, respectively, and 1,491 as “others”, whose miRNA regulation values are null (Table S7). We further investigated the distribution of miRNA regulation value, gene expression level and the potential relationship between these two parameters. In the 197 anti-correlated genes, 171 (account for 13.18% of 1, 297 down-regulated genes) were down-regulated and 26 (account for 4.48% of 581 up-regulated genes) were up-regulated. Gene distribution based on the regulation value also suggested that the anti-correlated group were always down-regulated by miRNAs (Figure 3A). In the 190 correlated genes, 140 (account for 10.79% of 1, 297 down-regulated genes) were down-regulated and 50 (account for 8.61% of 581 up-regulated genes) were up-regulated. Gene distribution based on the regulation value suggested that the correlated group were always down-regulated (Figure 3A). For 1,491 genes in “others”, 986 (account for 76.02% of 1,297 down-regulated genes) were down-regulated and 505 (account for 86.92% of 581 up-regulated genes) were up-regulated (Table 3). In conclusion, these results indicate that miRNAs tend to down-regulate gene expression, especially for those of the anti-correlated group. We also investigated potential functions of the anti-correlated group, which were supposed to be regulated by miRNAs, and found they were involved in a variety of biological processes, including metabolism, immune systems, cell killing, multicellular organismal development, and cell communication (Figure S3).

**Table 3:** Three groups of mRNAs predicted with different methods.

		miRanda*	TargetScan*	Conserved*
**Anti-correlated**	**All**	482	472	197
	**Up-regulated**	53(9.12%)	55(9.47%)	26(4.48%)
	**Down-regulated**	429(33.08%)	417(32.15%)	171(13.18%)
**Correlated**	**All**	294	285	190
	**Up-regulated**	117(20.14%)	108(18.59%)	50(8.61%)
	**Down-regulated**	177(13.65%)	177(13.65%)	140(10.97%)
**Others**	**All**	1102	1121	1491
	**Up-regulated**	411(70.74%)	418(71.94%)	505(86.92%)
	**Down-regulated**	691(53.28%)	703(54.2%)	986(76.02%)

*The proportion was calculated by dividing the number of all up-regulated mRNAs or down-regulated mRNAs.

We further verified this result using predictions of TargetScan without considering sequence conservation and predictions of miRanda. Using miRanda, we have 482 and 294 genes as anti-correlated and correlated, respectively, and 1,102 as “others”. Using TargetScan without considering conservation, we have 472 and 285 genes as the anti-correlated and correlated, respectively, and 1,121 as “others”. We further investigated the intersection between TargetScan and miRanda, and found that the three methods shared about 80% in the anti-correlated group; TargetScan and miRanda shared about 70% genes in the correlated group, while *P*_CT_ method shared only about 50% genes with the remaining two methods in the correlated group. Consistent with the results of conserved predictions, miRNAs in the anti-correlated group seem regulate higher proportion of down-regulated mRNAs as compared to the remaining groups defined based on the following two methods (Table 3), and the anti-correlated group exhibited strikingly different distribution of regulation value as compared to the correlated group (Figure 3A).

We then examined target expression level of the three regulation groups and found that expression level of the anti-correlated group was higher than the remaining groups (Figure 3B). Further investigation of regulation values with the expression level showed that highly-expressed genes in the anti-correlated group tended to be down-regulated by miRNAs, whereas lowly-expressed genes tended to be up-regulated. Genes in the correlated group behave in the opposite way (Figure 3C). This result suggested that highly expressed genes may contribute more to down-regulated genes in anti-correlated group than to up-regulated genes in the same group.

### Potential relationship of miRNAs with methylation in regulating mRNA expression

**Figure 4:**
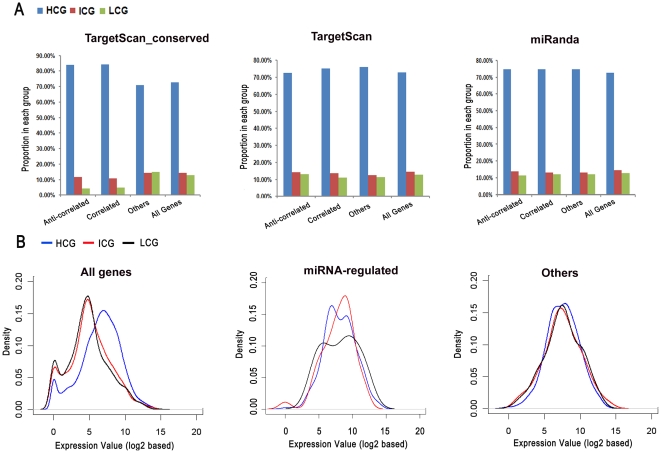
Potential relationship between miRNA and DNA methylation. (A) The distribution of CpG-density classified genes in each group. These target genes were predicted using three different methods: “Conserved” are those genes that have conserved miRNA binding sites among vertebrates or mammals, and these genes were predicted by using the *P*_CT_ method of TargetScan; “TargetScan” are those genes that are predicted by using a perl script of TargetScan without considering conservation. “miRanda” are those genes that are predicted potential targets based on miRanda v3.3a on Linux platform. (B) Relationship between CpG island density and gene expression. Three groups were classified: all expressed genes in NSCLC, the miRNA-regulated genes, and other genes which may not be influenced by miRNAs.

As methylation is another important mechanism to regulate mRNA expression, we further studied how it may interact with miRNA regulation. We first categorized the target genes into HCG (high CpG content), LCG (intermediate CpG content), and ICG (low CpG content) classes (Table S2 and Table S3) according a method previously described [35]. We found that in the conserved prediction results, both the anti-correlated and correlated groups contained more HCG genes than others or all genes (Figure 4A), and the result suggests that HCG genes are most likely to be regulated by miRNAs that have conserved target sites.

Since a large number of highly-expressed genes were found in the anti-correlated group, we further asked if these HCG genes are always highly-expressed and tend to miRNA-regulated. In all genes expressed in NSCLC, HCG genes were not seen as dominant in highly expressed genes as compared to LCG or ICG genes. The peak expressions of HCG range from 5 to 10, whereas those of ICG or LCG genes are in a range of 2 to 7. miRNA tends to regulate more genes at the relative expression level of 5 to 10, therefore, when regulated by miRNAs, the peak of ICG or LCG gene expressions should be in this range, and we observed that there were less LCG genes in this expression range as compared to HCG or ICG genes. However, “others” (genes with no regulation value variation) also has a peak in the expression range but there was no difference in gene density among the three CpG classes (Figure 4B). In summary, HCG genes may be enriched genes that are regulated by miRNAs due to their high expression levels, but this is obviously not the only reason.

**Figure 5:**
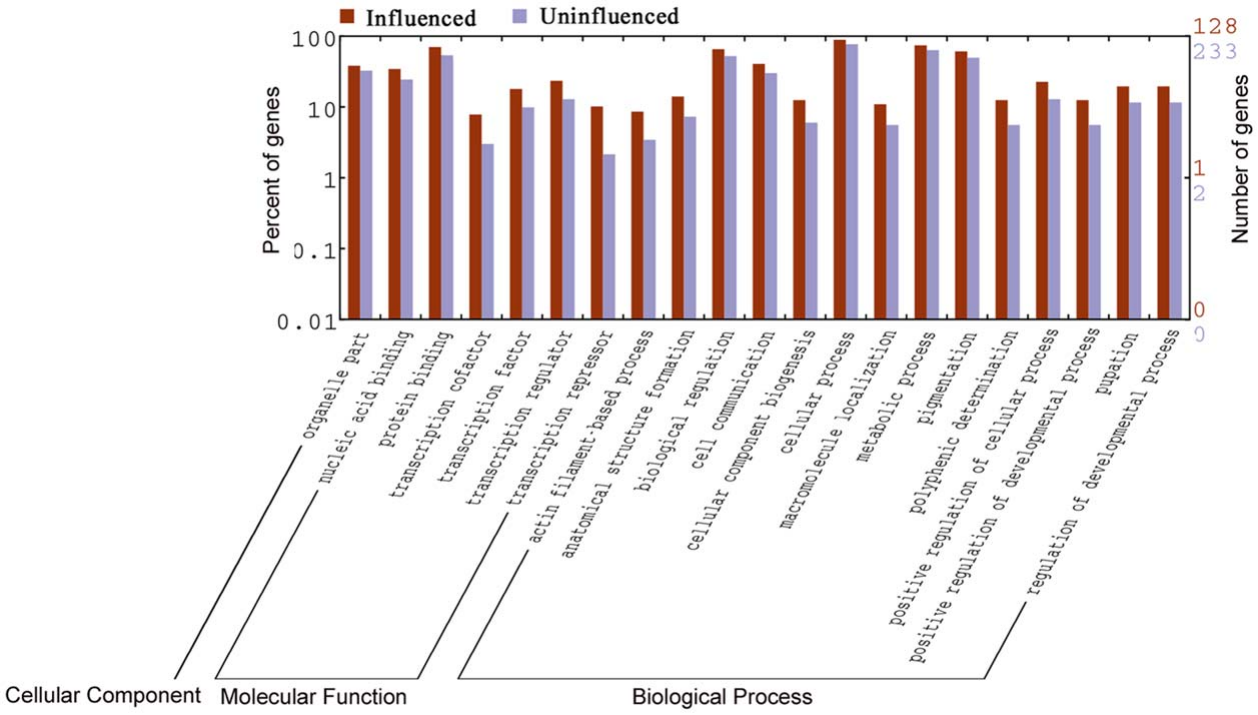
Different GO terms between the miRNA-regulated and miRNA-unregulated genes. *P*<0.05 was considered as significant [67].

We also performed GO analysis between the miRNA-regulated and miRNA-insensitive groups to show that their genes and functions are different from the anti-correlated and the correlated groups (Figure 5 and Figure S5). As it is shown in Figure 5, the miRNA-regulated group contains more genes in the following processes: transcription regulation, nucleic acid binding, cell communication, metabolic, and development regulation. These differences demonstrate from another angle why miRNAs tend to affect HCG genes with conserved miRNA target sites as CpG island content is always correlated with gene regulation and functional differentiation, and these genes may be of importance for basic cellular functions of different vertebrates or mammals [36].

## Discussion

In this study, we investigated the mRNA and miRNA profiling of NSCLC. mRNA profiling revealed that a number of differentially-expressed genes are involved in activation of cell cycle in NSCLC. This finding is consistent with results of previous studies, which suggest that abnormal cell cycle is associated with elevated lung cancer risk [37], [38]. The down-regulated genes are found to be predominantly enriched in immune systems as well as those localized on cell membranes, which are usually correlated with cell-cell communication and participate in immune process. Inhibitions of these genes have been supposed to facilitate lung cancer development and progression, and this may to some extent due to the escape of cancer cells from detection and destruction by the host immune system [39], [40], [41].

In miRNA profiling comparison, we found hsa-miR-96 was significantly up-regulated in NSCLC. 48 hsa-miR-96 potential targets were predominantly enriched in the down-regulated mRNA group and are involved in a variety of biological processes according to GO annotations. Based on regulation value estimates, we showed that 42 of them belong to the anti-correlated group (Figure 1A, Table S7). Our validation experiments revealed that the mature form of hsa-miR-96 is highly expressed not only in cancer tissues but also in sera of cancer patients. This result suggests that hsa-miR-96 has a great potential to be used as a noninvasive biomarker for NSCLC diagnosis.

Our current knowledge about hsa-miR-96 is rather limited. It resides in the intergenic area between NRF1 and UBE2H on human chromosome 7 [42] and has a very broad distribution among animals, from nematodes to mammals, and is more conserved among vertebrates (Figure S4). Previous studies have suggested that it functions to regulate the progression of differentiation in mammalian cochlear inner and outer hair cells [43], and is associated with aggressive human behaviors [44]. In the relationship with cancers, hsa-miR-96 has been reported to be highly expressed in bladder cancer [45], prostate carcinoma [46], and chronic myeloid leukemia [47]. It can be detected in urine and is correlated with stage and grade based on urinary cytology of urothelial carcinoma [48]. Up-regulation of hsa-miR-96 results in down-regulations of transcriptional factor FOXO3a and FOXO1, and thus induces cell proliferation in human breast cancer [49], [50]. In our assays, we found a set of potential targets that may correlate with the function of hsa-miR-96 in NSCLC development, but none of the potential targets have been validated by experiments. Therefore, further experimentation is of essence in revealing relationship between hsa-miR-96 and NSCLC. In addition, we will increase the size of the specimen to investigate possible relationship of hsa-miR-96 expression with prognosis, and other characteristics of NSCLC.

In General, miRNAs are believed to bind the 3′ untranslated region of a target mRNA and down-regulate its expression at mRNA or protein levels but mostly at mRNA level [51]. Therefore, when the expression of a miRNA is elevated, mRNAs that are regulated by the miRNA is expected to be down-regulated; when the expression of miRNA is repressed, its target mRNAs should show up-regulations. However, according to our results, the anti-correlated mRNAs are always down-regulated and only a small fraction of genes are up-regulated. Further examination suggested that in the anti-correlated group, down-regulated mRNAs are always highly expressed, and up-regulated mRNAs are always lowly expressed. Therefore, the existence of more highly expressed genes may be an important contributor to the effect. The reason why highly-expressed mRNAs tend to be down-regulated by miRNAs and the lowly-expressed mRNAs tend to be up-regulated by miRNAs suggests an existence of a regulatory balance that functions to keep the entire transcriptome in an optimized dynamic range.

In our analysis, we failed to find any clues that are responsible for the difference between the anti-correlate and the correlated mRNAs. GO classification did not show any obvious difference either (Figure S5). In addition, expression levels of the target mRNAs that have a high regulation value did not show significant variation, and vice versa (Table S2, Table S3,Table S7, Table S9, Table S11), and genes whose expression are significantly varied are not necessarily significantly regulated. Since a mRNA is often regulated by more than one miRNAs [28], we are still unable to know how these miRNAs interact with one another, and which kind of direct interactions are most effective. Furthermore, miRNAs may repress their target genes only at the protein level [52] and the targets may not exhibit noticeable changes at the mRNA level. There are other factors that are involved in regulating mRNAs at epigenetic levels, such as histone methylation [53], [54] and acetylation [55], [56], and that are always capable of interfering with miRNA-centric networks [57], [58], making deciphering such networks more difficult.

Another interesting observation is that genes in either the correlated group or the anti-correlated group have a larger proportion of HCG genes than that in “others” group. Both the correlated and the anti-correlated groups are considered most likely to be influenced or possibly regulated by miRNAs. This result suggests that miRNAs tend to regulate HCG genes. Further investigation demonstrated that HCG genes tended to express at a higher level when compared with ICG or LCG genes. Therefore, there may be a possibility that more HCG genes are influenced by miRNAs. However, comparisons based on all potential targets failed to show any difference among these groups (Figure 4A). As the stringent standard selected targets have conserved binding sites of miRNAs among vertebrates or mammals, more HCG target genes in miRNA-influenced group may correlate with gene evolution among vertebrates.

GO comparison between the regulated and the unregulated groups demonstrated they were quite different as the former always contains more genes involved in transcription regulation, nucleic acid binding, cell communication, metabolic, and development regulation. The function of genes and their expression is always correlated with the CpG island content of promoters. It has been suggested that house-keeping functions are significantly overrepresented in the HCG class, whereas terms associated with specific functions characteristic of more differentiated or highly regulated cells are significantly overrepresented in the LCG class [36], [59]. miRNA has been proposed to be a primary regulation mechanism as it is present from low to high organisms, it therefore may prefer to regulate a higher proportion of HCG genes for basic cellular functions. These are possible reasons why miRNA regulation may be more biased toward HCG genes.

DNA methylation plays a role in the repression of gene expression in animal cells, but in many cells, most genes are inactive even their CpG island-containing promoters remain unmethylated [35], [60]. This implies that there may be other regulatory mechanisms involved, such as histone modifications and miRNAs. Inactive HCG genes were more frequently unmathylated as compared with ICG or LCG genes, and it was most likely that the role of enriched demethylated H3K4 is to protect CpG islands from methylation [35]. In addition, a broad H3 hyperacetylation in CpG islands has been reported [61]. Therefore, histone modifications are most likely to affect DNA methylation regulated gene repression, and an increasing number of evidence has confirmed this conclusion [62], [63]. Lung cancer development is closely correlated with three most studied epigenetic phenomena including modifications in DNA and histone proteins as well as miRNAs [64]. The interaction between DNA methylation and histone modification have been well studied [65], while the relationship between DNA methylation and miRNAs or miRNAs and histone modification has not been elucidated. According to our results, there may be a possibility that miRNAs play a role in bridging DNA methylation repression and histone modifications.

## Materials and Methods

### Ethics Statement

All samples were obtained from the tissue bank of Zhoushan Hospital (city of Zhoushan, Zhejiang province, China), which assures written informed consent from all subjects. The Institutional Review Board of Zhoushan Hospital approved the use of samples for this study.

### Patients and RNA extraction

We performed miRNA microarray and mRNA microarray using 3 adenocarcinomas and 3 squamous cell carcinomas of the lung as well as the paired control samples from their adjacent normal tissues, which were carefully diagnosed and pathologically defined (Table S1). Specimens were brought to pathologists immediately for diagnosis (Figure S1). Tumor tissues and the corresponding adjacent normal tissues were placed in different tubes in liquid nitrogen and subsequently stored at -180°C. The Total RNA that was used to perform microarray analysis was extracted using TRIZOL (Invitrogen).

Since we used 3 adenocarcinomas (and controls) and 3 squamous cell carcinomas (and controls) of the lung for the microarray analysis, we chose a comparable number of corresponding NSCLC cases for validation. We also took into consideration of the coverage of different genders, cancer stages, ages, and other characteristics. In particular, we performed tissue qRT-PCR validation using 35 pair specimens (17 paired samples of adenocarcinomas and 18 paired samples of squamous cell carcinomas of the lung), and further examined miRNA expression abundance in the serum samples from the same 35 specimens. There are 17 patients whose ages are more than 60 and 18 younger than 60 (including 7 males and 28 females). As for the grade of differentiation, the tumors are 13 well differentiated, 18 moderately differentiated, and 4 are poorly differentiated. The tumor stages are also clearly defined: 28 are at T1-T2 and 7 are in T3-T4. In addition, lymph node metastases were found in 15 patients (8 patients are at stage I and 17 patients are higher stages; Table S1). We collected the sera before surgical resection and chemotherapy. We selected 20 people (9 males and 11 females; all aged below 60) without any cancer histories and other illness and collected their sera as normal controls. Total RNA including miRNA from the tissue and serum samples were extracted by using a commercial kit (mirVana RNA™ Isolation kit, Applied Biosystems) according to the supplier's instruction. Quality of total RNA was determined by using Bioanalyzer (UV spectrophotometer Q3000, Quawell). Extracted RNA samples were stored at -80°C until used.

For mRNA expression validation, we used 20 pair specimens (9 paired samples of adenocarcinomas and 11 paired samples of squamous cell carcinomas of the lung. 12 pair were from the previous 35 patient cohort.). In particular, There are 5 patients whose ages are more than 60 and 15 patients who are younger than 60 (17 male and 3 female). For the grade of differentiation, 11 and 9 are moderately differentiated and poorly differentiated, respectively. For tumor stages, 19 patients are at T1-T2 and one is at T3-T4. Lymph node metastases were found in 5 patients. For the tumor stage, 9 patients have stage I and 11 patients have higher stage tumors (Table S1).

### Quantitative real-time PCR

10 ng of total RNA was reverse-transcribed using the TaqMan miRNA reverse transcription kit and RT primers for miR-96 and U6 snRNA (Applied Biosystems). The cDNAs were then analyzed by real-time PCR using TaqMan probes for miR-96, and U6 snRNA (Applied Biosystems). We reverse transcribed three micrograms of total RNA to single-stranded cDNA using Fermentas kit and then performed qRT-PCR experiments using SYBR Green (RealMasterMix(SYBR Green), TIANGEN) on Applied Biosystems 7500 Real-Time PCR System. We analyzed gene expression using 2^ΔΔCT^ method [66]. Relative expression of hsa-miR-96 was determined in reference to an internal U6 snRNA control, and relative mRNA expression was determined in reference to glyceraldehyde-3-phosphate dehydrogenase (GAPDH). We listed the primer sequences that were used in mRNA expression validation experiments in Table S12. In the statistical analysis, we presented results as mean ± SE, and assessed differences between the groups by using paired or independent T-Test in SPSS 15.0.

### Microarray hybridization

mRNA profiling were performed by using Illuminia Technologies “HumanHT-12 v4” according to manufacturer’s protocol. miRNA profiling were performed by using Illuminia Technologies “humanMI_V2” according to the manufacturer's protocol. GenomeStudio 1.0 was used to perform average normalization of the results from mRNA and miRNA microarrays. All data is MIAME compliant and that the raw data has been deposited in a MIAME compliant database. The expression data generated by this study are available in the NCBI Gene Expression Omnibus (GEO) as accession GSE29250.

### Differential expression analysis

In each group-based comparison, we filtered out all miRNAs or mRNAs that were not detected in any sample. Considering that these samples may be too few to draw any reliable conclusions about differential expression, we only chose genes that are consistently up- or down-regulated in all the 6 NSCLC tissues compared with the adjacent normal tissues as the differential expression results. To get the significantly differential expression results, the signal values were transformed (log2) and median centered for each array, and then two-class paired differential expression analysis was performed by using SAM (version 3.11; Stanford University). Genes with significant differences were selected at a FDR of 0.1.

### miRNA target prediction and miRNA regulation value

We used three methods to predict miRNA targets. UTR sequences were downloaded from the website of TargetScan. We used perl scripts of TargetScan to predict targets. First, we predicted targets with conserved miRNA binding sites using the *P*_CT_ method of TargetScan [33]. We then predicted all the potential targets using TargetScan without considering sequence conservation. At last, we run miRanda v3.3a on Linux platform as the third method to predict potential targets using default parameter settings.

We hypothesized that the average regulation value of a miRNA is correlated with its expression level and the number of targets, and can be calculated as: the expression value variation divided by the number of expressed targets. During the process of cancer development, some miRNAs may be up-regulated in cancer as compared with the adjacent normal tissue, and in contrast, some may be down-regulated, and therefore the regulation value will become positive or negative respectively if the number of its expressed targets is not changed significantly. For an mRNA, it can be regulated by a variety of miRNAs, so the regulation value is calculated by the addition of regulation value variations over all variable miRNAs.

## Supporting Information

Figure S1 Histological images of lung tumor tissues and the adjacent normal lung tissues.
(TIF)

Figure S2 GO results of the 48 down-regulated conserved targets of hsa-miR-96 (predicted using TargetScan).C
(TIF)

Figure S3 GO results of the anti-correlated genes (predicted using TargetScan, and the conserved target sites were chosen).
(TIF)

Figure S4 Mature sequence alignment of hsa-miR-96 in different species.
(TIF)

Figure S5 Different GO terms between the anti-correlated group and the correlated group.
(TIF)

Table S1 Information for the 35 patients that were used in qRT-PCR examination and the 6 patients that were used in performing microarray.
(XLS)

Table S2 Up-regulated genes in NSCLC cancer tissue compared with the adjacent normal tissue.
(XLS)

Table S3 Down-regulated genes in NSCLC cancer tissue compared with the adjacent normal tissue.
(XLS)

Table S4 Up-regulated miRNAs in NSCLC cancer tissue compared with the adjacent normal tissue.
(XLS)

Table S5 Down-regulated miRNAs in NSCLC cancer tissue compared with the adjacent normal tissue.
(XLS)

Table S6 Target genes (predicted using *P*_CT_ method of TargetScan) which have conserved miRNA binding sites among vertebrates or mannals.
(XLS)

Table S7 Target genes which have conserved miRNA binding sites.
(XLS)

Table S8 All potential target genes that are predicted by TargetScan.
(XLS)

Table S9 Regulation pattern based on all potential target genes that are predicted by TargetScan.
(XLS)

Table S10 All potential target genes that are predicted by miRanda.
(XLS)

Table S11 Regulation pattern based on all potential target genes that are predicted by miRanda.
(XLS)

Table S12 Primer sequences for mRNA expression validation experiments.
(XLS)
