# Supplementary figures and images for: An Integrated Analysis of miRNA and mRNA Expressions in Non-Small Cell Lung Cancers
Source: PLoS One. 2011 Oct 27;6(10):e26502. doi: 10.1371/journal.pone.0026502 (PMC3203153; doi:10.1371/journal.pone.0026502)

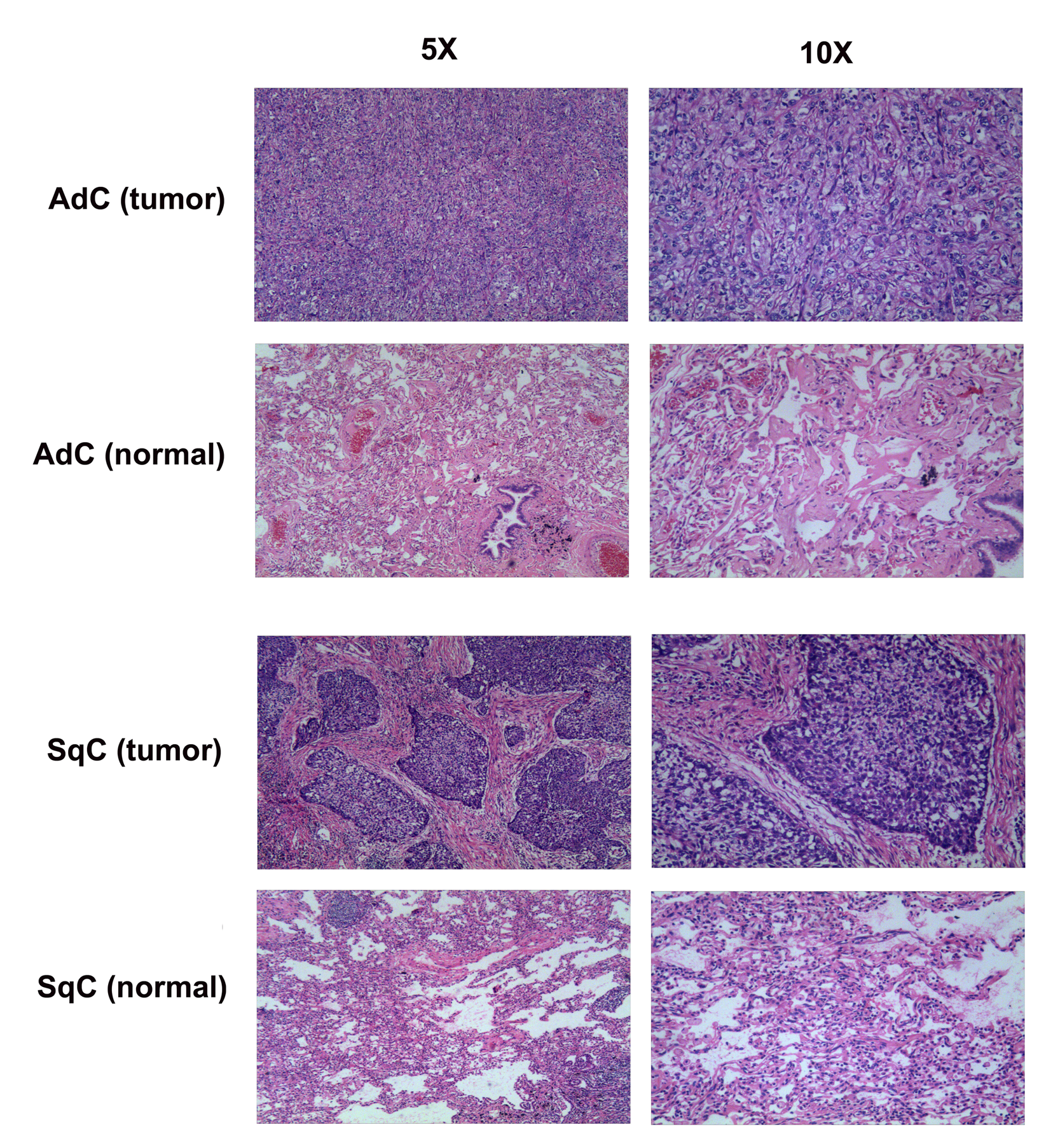

Supplement: Figure S1 — Histological images of lung tumor tissues and the adjacent normal lung tissues. (TIF) [file pone.0026502.s001.tif]

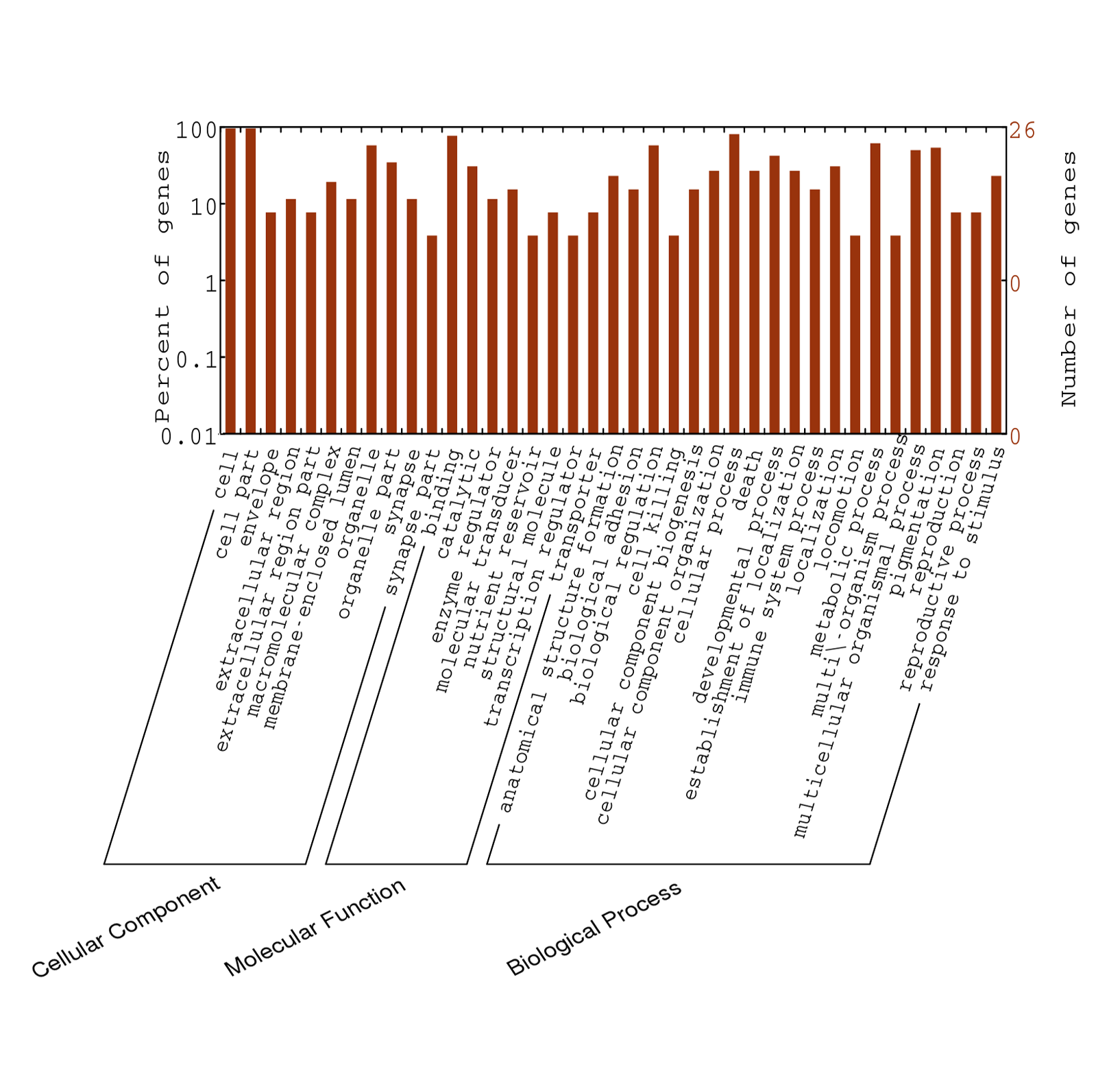

Supplement: Figure S2 — GO results of the 48 down-regulated conserved targets of hsa-miR-96 (predicted using TargetScan).C (TIF) [file pone.0026502.s002.tif]

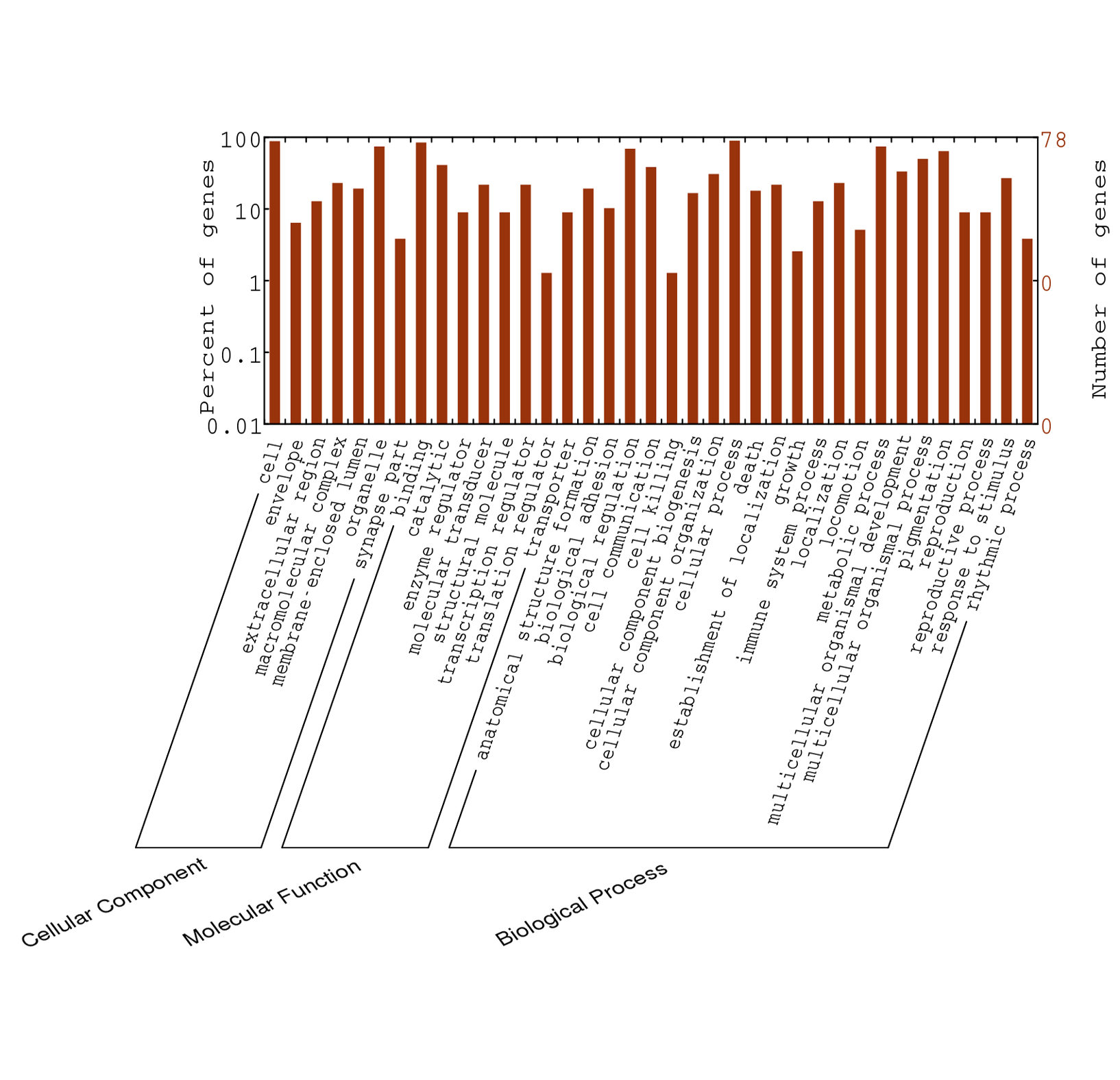

Supplement: Figure S3 — GO results of the anti-correlated genes (predicted using TargetScan, and the conserved target sites were chosen). (TIF) [file pone.0026502.s003.tif]

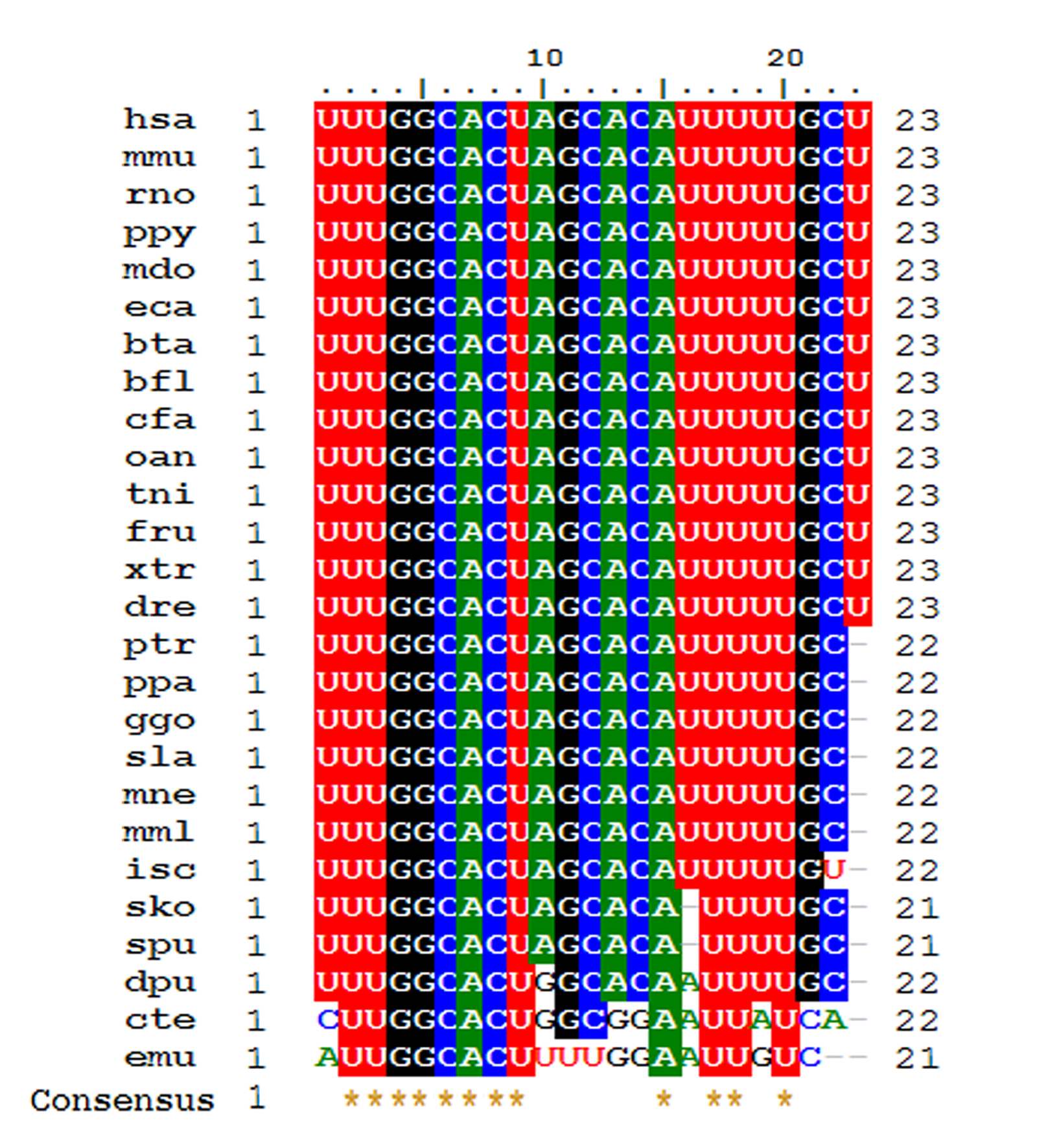

Supplement: Figure S4 — Mature sequence alignment of hsa-miR-96 in different species. (TIF) [file pone.0026502.s004.tif]

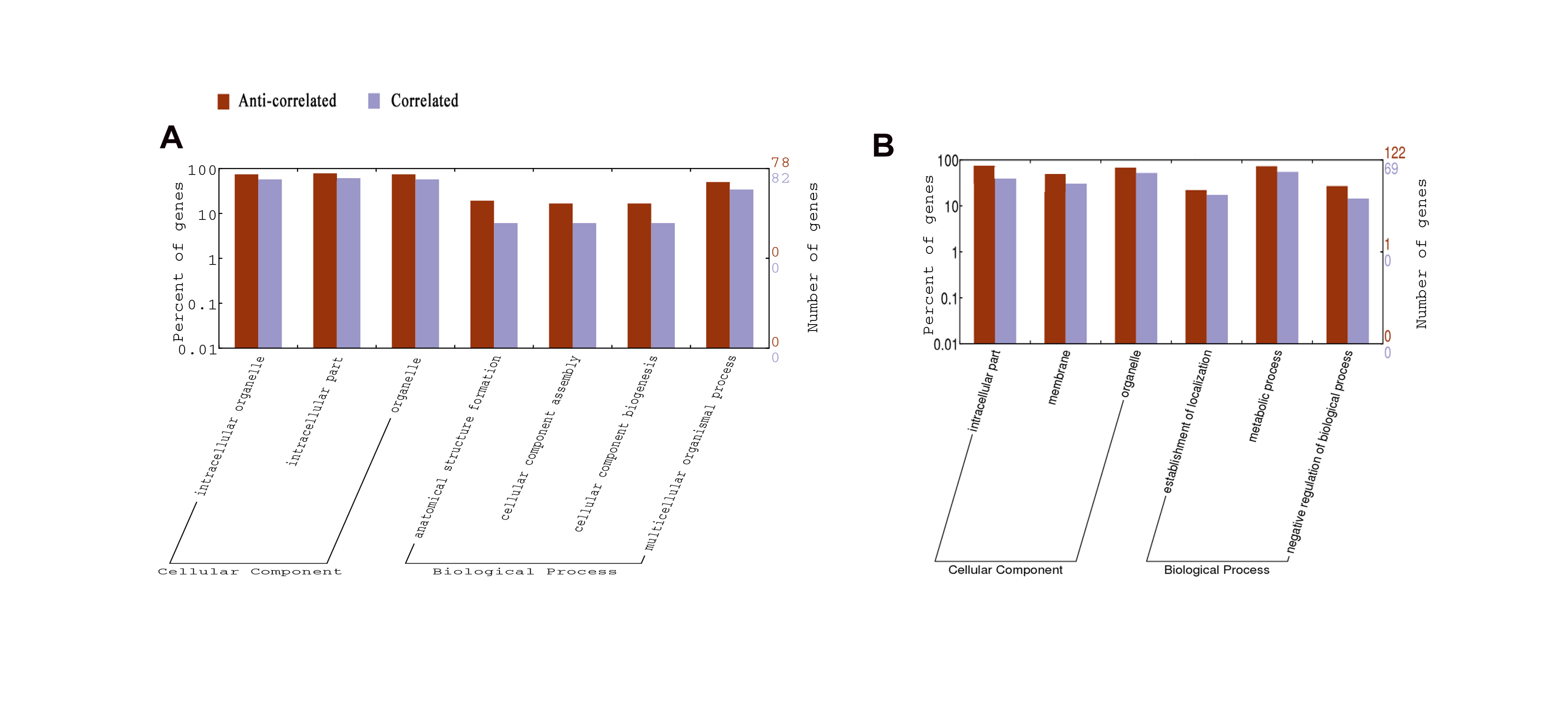

Supplement: Figure S5 — Different GO terms between the anti-correlated group and the correlated group. (TIF) [file pone.0026502.s005.tif]
